# Social Psychiatry in the Waiting Room: What a Physician Can Learn about Occupational Stress from Workers Waiting to Be Examined

**DOI:** 10.1155/2013/701872

**Published:** 2013-03-06

**Authors:** Nicola Magnavita, Sergio Garbarino

**Affiliations:** ^1^Department of Public Health, Catholic University School of Medicine, Largo A. Gemelli 8, 00168 Rome, Italy; ^2^State Police Health Service Department, Ministry of the Interior, 00100 Rome, Italy; ^3^Department of Neuroscience, Ophthalmology and Genetics, University of Genoa, 16126 Genoa, Italy

## Abstract

*Background*. Work-related stress is a major problem for mental health. The occupational physician has the opportunity to gather information on the perception of stress from workers in the course of regular medical examinations. *Method*. 1,231 subjects, engaged in 6 different occupations, were invited to compile the Demand/Control/Support and the Effort/Reward/Imbalance questionnaires. *Results*. A specific profile of work-related stress emerged for each group of workers. Radiology physicians reported high control over work, but also exceedingly high demand and effort, high overcommitment, low social support, and low rewards from work. Health care workers were often overcommitted but had high levels of reward and social support. Low levels of social support and reward were recorded for mature workers, while special force policemen engaged in law enforcement during the G8 meeting had high levels of social support and regards, so that their resulting stress levels were closer to the reference group of employees in an insurance company with no front-office. *Conclusion*. The practice of administering questionnaires to groups of workers who are subject to medical surveillance is useful for monitoring mental health and well-being.

## 1. Introduction

Traditionally, health surveillance has been defined as “the periodic medicophysiological examination of exposed workers with the objective of protecting and preventing occupationally related diseases” [1]. It includes “any procedure undertaken in individuals or groups to review an employee's health and assess any significant deviation from normality” [2]. Health surveillance is indicated when there is a continuing potential for occupational exposure to a hazard and there is a valid method of surveillance with adequate means for interpreting the findings [3].

In European countries, health surveillance is compulsory. Occupational physicians must collect the most useful data relating to the health and well-being of a large number of workers over a specific period of time.

Work-related stress is considered a major risk in all categories of workers. The socioepidemiological approach, frequently used to explain the causes of work-related stress, suggests that certain work characteristics increase the susceptibility of the worker to the risk of distress, with negative consequences for mental and physical health. Although there are a number of ways in which the psychosocial work environment can be assessed, self-reported questionnaires are the commonest type of psychosocial hazard measurement. 

Different and competing conceptual models have been proposed to investigate the relationship between job characteristics and stress. Among these, the two leading models claimed to have the most explanatory power are the Job Demand/Control/Support (DCS) model, developed by Karasek [4], and the Effort/Reward Imbalance (ERI) model, developed by Siegrist [5]. 

The DCS model assumes that the primary sources of job stress lie within two basic characteristics of the job itself: “job demand” and “job control.” This model predicts that job strain is not simply a function of job demand but also depends on the amount of control the worker has over the work. Job demand takes into consideration the pace and intensity of work: that is, work overload, degree of difficulty, available time, time allotted to executing tasks, and the existence of contradictory or conflicting orders. Job decision latitude, or job control, depends upon the worker's ability to control his own activities and skill usage. Social support at work, a moderator factor, was later included in the model [6]. Much of current job stress research has been based on this model [7–11]. 

The ERI, an alternative theoretical model, emphasizes the reward rather than the control structure of work, suggesting that mental distress and its health effects arise when a high degree of effort is not adequately rewarded in the form of pay, esteem, status consistency, or career opportunities. A further assumption of this model concerns individual differences in the experience of effort-reward imbalance; people characterized by a motivational pattern of excessive work-related commitment and high need for approval (overcommitment) are at increased risk of strain [12]. The number of published empirical studies based on the ERI model is growing rapidly, and the combination of high effort and low reward at work has been found to be a risk factor for many physical and psychological diseases [13–17].

Although both models have made distinct contributions to explaining perceived work stress in different types of occupations [18], a combination of the two models has been shown to improve our understanding of the relationship between the psychosocial work environment and workers' health [19].

The purpose of this paper is to illustrate the results that can be obtained by measuring the perception of stress among workers using both the DCS and ERI models simultaneously.

## 2. Subjects and Methods

We studied a total of 1,231 subjects, belonging to 6 groups of workers: (a) radiologists and radiotherapists, 314 subjects (251 males, 80%; 63 females, 20%); (b) health care workers in a hospital specializing in infectious diseases, 217 subjects (94 males, 43%; 53 females, 57%); (c) health care workers in a general hospital, 162 subjects (58 males, 36%; 104 females, 64%); (d) the employees of an insurance company with no front-office, 51 subjects (19 males, 37%; 32 females, 67%); (e) mature workers (>50 years) from a provider of social services, 197 subjects (126 males, 64%; 71 females, 36%); (f) policemen engaged in law and order operations during the 2009 G8 meeting in Italy (290 male subjects). Workers were invited to complete the questionnaires while awaiting medical examination. The response rate ranged from 84% in the general hospital workers to 99% in policemen. The study was approved by the Ethics Committee of the Catholic University of the Sacred Heart, Rome, Italy.

Table 1 contains the personal data of the study population.

Both DCS and ERI questionnaires are available in different versions that are theoretically consistent, despite differences in the wording of questions and response formats [20–22]. In this study, we adopted the 17-item version of the DCS questionnaire and the 23-item version of the ERI questionnaire. Validation of the translation ensured that the Italian version maintained the characteristics of the original and that the internal reliability (Cronbach's alpha) of each subscale was satisfactory [23].

The classic 17-item DCS questionnaire consisted of 3 scales termed “psychological job demand” (D), “job control or decision latitude” (C), and “workplace social support” (S). The “demand” scale was the sum of 5 items (e.g., D1: “Do you have to work very fast in your job?”) (alpha reliability was 0.76); the “control” scale was the sum of 6 items (e.g., C1: “Do you have the opportunity to learn new things in your work?”) (alpha = 0.67); the “support” scale was the sum of 6 items (e.g., S1: “There is a calm and pleasant atmosphere where I work”) (alpha = 0.87). Items were scored using a 4-point Likert scale in which the first two scales were graded from 1 = never to 4 = often, while the third scale (support) was graded from 1 = strong disagreement to 4 = strong agreement. We followed the commonest method of obtaining a continuous variable, termed “perceived job strain,” and divided demand by control (weighted by item numbers). Thus, the score from the three scales ranged from 5 to 20 points for the D scale and from 6 to 24 for the C and S scales. Job Strain (JS) was calculated as the weighted ratio between the scores on the D and C scales. A participant was defined as having high job strain if he/she scored high on job demands and low on job control (defined as above the median score on the respective scales). Participants who reported low levels of social support (median split) together with job strain (high job demands and low job control) were defined as having high isostrain. 

The 23-item ERI questionnaire contained two scales: “effort,” evaluated by 6 items (e.g., E1 “I have constant time pressure due to a heavy workload”) and “reward,” evaluated by 11 items (e.g., R1 “I receive the respect I deserve from my superior or equivalent person”). Both were scored on a 5-point scale, where a value of 1 indicated no stressful experience and 5 indicated a highly stressful experience. The weighted ratio between effort and reward was calculated to quantify the degree of mismatch between effort and reward. The ERI questionnaire also included a third scale, “over-commitment,” which was evaluated by 6 items on a 4-point Likert scale (e.g., O3: “When I get home, I can easily relax and “switch off” work”). It measured the set of intrinsic personal factors regarding occupational motivation and participation that enhance the effects of stress. Cronbach's alpha values of the three sub-scales were: 0.89, 0.88, and 0.89. Consequently, the score for sub-scale E ranged from 6 to 30 points, that for sub-scale R from 11 to 55 points, and the sub-scale O score ranged from 6 to 24 points. The effort/reward imbalance was calculated as the weighted ratio of reward and effort. A participant was defined as having E/R imbalance if he/she scored high on the effort scale and low on the reward scale (defined as above the median score on the respective scales). Participants who reported high levels of overcommitment (higher tertile) together with E/R imbalance (high effort and low reward) were defined as having E/R imbalance and over-commitment. 

### 2.1. Statistics

The average values of stress-related variables were compared using general linear methods, adjusted for sex and age. The prevalence of cases of strain, isostrain, and imbalance in the different groups was compared by Pearson's chi square. Logistic regression analysis was used to calculate the odds ratio (OR) and confidence interval at 95% (95% CI) of the occurrence of job strain and effort/reward imbalance in each category of workers. Employees in the insurance company with no front-office was chosen as the reference group.

Data were analyzed by the Statistical Package for Social Sciences PASW/SPSS 20.0 (IBM, Chicago, IL).

## 3. Results

Both models revealed a highly significant disparity in work stress among the different groups (*P* < 0.0001) (Table 2).

Demand was very high among radiologists (17.0 ± 1.6) and low in insurance employees (11.8 ± 2.8). Control over work was high in radiologists (19.8 ± 2.5) and health care workers from the specialized hospital (19.6 ± 2.5) and low in the general hospital workers, police, and mature workers (range 16.4–16.6). Social support was high in hospital workers from both the general hospital and the hospital for infectious diseases (20.3 ± 3.4) and low in mature workers (18.2 ± 4.3) and radiologists (18.3 + 3.8). Perceived stress, measured using Karasek's model as a weighted ratio between work demand and control was highest in the mature workers and the radiologists (both groups had a mean score = 1.05) and lowest in health care workers from the hospital for infectious diseases (0.86 + 0.18) and insurance company employees (0.84 ± 0.28). The percentages of workers in the “high risk” category of job strain, that is, high demand and low control, were highest in radiologists and general hospital workers (28.7% and 30.4%, resp.). Among health care workers in the general hospital, there was also a significant proportion of subjects affected by social isolation due to job strain (21 cases, 14.9% of the population).

Using the Siegrist model that defines stress as the difference between effort spent and rewards obtained, we found that effort was highest among radiologists (17.8 ± 5.6) and lowest among insurance clerks (11.3 ± 3.5), while reward was highest in policemen (45.6 ± 7.4) and lowest in radiologists (42.1 ± 8.6). Consequently, the group with the highest occupational stress was that of the radiologists (ERI = 0.87 ± 0.50), followed at some distance by the mature workers (0.71 ± 0.36) and the general hospital health care workers (0.69 ± 0.46). The latter were followed by health care workers from the hospital for infectious diseases (0.55 ± 0.38), the G8 policemen (0.52 ± 0.29), and finally the clerks (0.48 ± 0.18) who had the lowest stress level. The highest percentages of workers with high effort/reward imbalance were in the group of radiologists (47.1%), followed by mature workers (42.9%). The insurance company employees had a very low percentage of “imbalanced” subjects (7.8%). Over-commitment, which calculates, by means of Siegrist's model, individual internal effort and therefore personal tendency to suffer the negative effects of stress, was highest in all groups of health care workers: radiologists (9.8 ± 4.7), general hospital workers (8.2 ± 3.2), and workers from the hospital for infectious diseases (7.4 ± 3.0). A low level of over-commitment (very close to the lowest value theoretically possible) was found in policemen (6.6 ± 1.8).

Logistic regression analysis showed that compared to the clerks, the radiologists had a more than 6 times greater risk of having abnormal job strain and a 12-fold increased risk of effort/reward imbalance. Even workers in the general hospital and mature workers showed significant increases odds for both DCS and ERI distress cases (Table 3).

## 4. Discussion

Our study demonstrates that the physician can easily obtain a substantial amount of information from workers awaiting medical examination. Self-compiled questionnaires can be completed in the waiting room without subtracting any time from medical examinations and at a negligible cost. The use of a laptop may be helpful for avoiding errors of distraction (e.g., skipping a question) and any mistakes made during the transcription of answers [24]. Participation is not compulsory and does not influence the physician's judgment regarding occupational fitness. Workers willingly participate in the survey, because they know that their responses are confidential and the collective results can be used only in their interest. Analysis of the results is simple and provides useful information for risk prevention in the categories of workers examined, as well as giving the physician the opportunity to identify distressed workers.

The practice of administering questionnaires to groups of workers who are subject to medical surveillance is to be recommended for four reasons. The first is that it enables us to understand the profile of each profession and to obtain information that would otherwise be very difficult to collect in the course of medical examinations. The second is that the comparison between different groups of workers is the simplest way to obtain indications concerning the level of health risk associated with work-related stress. Although they are widely used for many decades, the DCS and ERI questionnaires do not have a threshold limit value. In published studies, the researchers simply divide the observed values in percentiles and compare subjects with the highest values and those with the lowest values. To our knowledge, this is the first study in which groups of Italian workers were compared using both the DCS and ERI questionnaires in order to ascertain which job causes the highest levels of work-related stress. Thirdly, the simultaneous use of two questionnaires makes it possible to compare the two models of stress. Finally, we show that the job of law enforcement, that is automatically considered to be very hazardous, rarely causes an increase in stress levels in workers.

Although the two most frequently used stress models are closely correlated from a statistical point of view, they provide complementary rather than comparable results. In studies of occupational stress, replacing one model by another may change the observed effects considerably, and may have different implications for policy; whereas the control paradigm refers to the division of labour, the reward paradigm addresses the issue of distributive justice and fairness. Components of the extrinsic part of the ERI model (salaries, career opportunities, and job security) are linked to macroeconomic market conditions, while the Karasek model focuses on workplace characteristics [12]. Thus, the job strain model is probably a better tool for the objective measurement of stressors, while the effort-reward model deals more with cognitive levels of perceived job stress [25]. The simultaneous use of both models may be useful as each of the models captures different aspects of occupational stress and associations with health effects appear to be independent of each other [26]. Nevertheless, few investigations have analyzed the psychometric properties of both scales when applied simultaneously [27], and little research has been carried out to determine which kind of model would best fit a specific situation.

Differences in the two methods help us to distinguish the varying characteristics of job stress in different types of occupation. 

Surprisingly, each of the two study methods demonstrated that the radiologists, who are seen as belonging to a privileged category on account of their substantial salary and highly technological work performed in a controlled environment, had the highest mean levels of stress and the highest percentage of individuals at risk. In fact, the specific characteristics required for diagnosis by imaging, the limited patient contact (and subsequent reduced social support), high degree of responsibility, and workload that cannot be managed by the person involved, provide a rational explanation for the “strain” measured by DCS model, while the disparity between the high degree of professional commitment and the static nature of material and immaterial rewards offered by the health service in subjects who are naturally prone to professional overcommitment explains the change in stress scores measured by the ERI model. Previous studies have identified specific stressors in the work of radiologists and radiotherapists [28–33] and have shown that the latter often derive little satisfaction from their job [31, 34].

Mature employees in social service activities are particularly affected by physical work factors that can be measured more efficiently by means of the Karasek questionnaire. The very fact that they are still active in spite of their age demonstrates that they often have a positive perception of the balance between what they give in their work and what they receive in exchange, and this explains a lower stress score by the Siegrist model. Although they have low rewards, they also make low efforts at work. Social isolation, which is typical of a mature age, was reflected in the low levels of social support. Similar low values were found for radiologists who, in fact, work alone. These findings are in agreement with the literature. Studies across countries and continents show that psychosocial stress at work may be a relevant risk factor for depressive symptoms among older employees [35]. Older workers in high-stress jobs may be at increased risk of occupational stress-related health problems, especially if they try to cope with stress by adopting behavior that puts their health at risk [36, 37].

As expected, health care workers from the two hospitals were found to have a similar stress level profile. Psychosocial problems in health care activities are well known and widespread [38–42]. However, work in a highly specialized environment such as that of the hospital for infectious diseases is more highly qualified and therefore more rewarding and less tedious than in a general hospital, with consequent benefits for both the JCQ and ERI stress levels. All health care workers, including the radiologists, had higher average over-commitment scores than other categories of workers. This finding is not surprising, since subjects who choose the caring professions commit their personal resources beyond what is reasonable and react to problems with a greater personal commitment. On the contrary, the elite team of policemen who were continuously engaged in antiriot operations and law and order enforcement are specifically trained to avoid getting emotionally involved by the events and thus have levels of over-commitment that are close to zero.

The police engaged in law and order operations had stress levels that appeared to be elevated when measured by the Karasek model which takes into consideration the high workload and very limited discretionary power in a hierarchic structure. However, these levels seemed moderate when determined by the Siegrist model since the latter highlighted the fact that these policemen belonged to an elite population who were used to elevated levels of effort and who obtained a high degree of immaterial reward as a result of successful law-keeping operations during the G8 meeting. It must be remembered that this survey was carried out shortly before the G8 meeting where policing operations were seen to be highly successful. Studies conducted during routine law enforcement revealed that police officers were often exposed to higher levels of stress than those reported for the G8 event [43].

Regardless of the model employed, clerks had the lowest stress levels.

This study has some limits due to the fact that it was a field study that could not randomize observations or layer the populations. The results should be examined with great caution, because it is a descriptive study. Before generalizing our findings, one must perform a careful analysis of the socio-demographic and environmental factors and take account of confounders. What we have shown is only the first step of the work. The occupational physician then has the task of analyzing with stringent epidemiological criteria the data, in order to propose prevention programs, if necessary. Further studies are needed to ascertain whether the differences observed reflect real variations in stress levels that are attributable to different types of occupations. However, it seems likely that the models, or some of their psychosocial aspects, provide important information for explaining perceived stress in a number of different occupations.

## Figures and Tables

**Table 1:** Characteristics of the observed samples.

Population	Number	Gender, male *N* (%)	Age (mean ± s.d.)
A-radiologists	314	251 (79.9)	48.2 ± 8.3
B-health care workers, infectious diseases hospital	217	94 (43.3)	40.1 ± 8.9
C-health care workers, general hospital	162	58 (35.8)	43.2 ± 9.1
D-insurance company white collar workers	51	19 (37.3)	40.1 ± 7.0
E-elderly workers	197	126 (64.0)	56.5 ± 7.0
F-policemen	290	290 (100.0)	35.4 ± 7.5

Total	1231	838 (68.1)	44.1 ± 10.8

**Table 2:** Score of the stress-related variables (mean ± standard deviation) and prevalence of workers in the high-risk category.

	A-radiologists		B-infectious diseases hospital		C-general hospital		D-insurance		E-mature		F-police		*P*
	Mean	s.d.	Mean	s.d.	Mean	s.d.	Mean	s.d.	Mean	s.d.	Mean	s.d.	
Demand (range 5–20)	17.0	1.6	13.7	2.3	13.2	3.0	11.8	2.8	13.0	3.0	12.6	2.7	<0.001^b^
Control (range 6–24)	19.8	2.5	19.6	2.5	16.6	3.0	17.6	3.1	16.4	4.4	16.6	4.1	<0.001^b^
Support (range 6–24)	18.3	3.8	20.3	3.4	19.7	3.6	19.1	2.9	18.2	4.3	19.0	3.1	<0.001^b^
D/C ratio	1.05	0.18	0.86	0.18	1.00	0.30	0.84	0.28	1.05	0.48	0.99	0.40	<0.001^b^
Job strain, N (%)	90 (28.7)		28 (12.9)		45 (30.4)		4 (7.8)		36 (19.9)		48 (16.6)		<0.001^a^
Isostrain, N (%)	24 (7.6)		13 (6.0)		21 (14.9)		2 (3.9)		12 (6.9)		20 (6.9)		<0.05^a^
Effort (range 6–30)	17.9	5.6	12.3	3.8	14.6	5.0	11.3	3.5	13.8	4.8	11.8	3.8	<0.001^b^
Reward (range 11–55)	42.1	8.6	45.3	8.1	45.5	9.3	44.9	8.3	39.0	8.5	45.6	7.4	<0.001^b^
Over-commitment (range 6–24)	9.8	4.7	7.4	3.0	8.2	3.2	7.1	2.0	n.e.	n.e.	6.6	1.8	<0.001^b^
ERI	0.87	0.50	0.55	0.38	0.69	0.46	0.48	0.18	0.71	0.36	0.52	0.29	<0.001^b^
ERI, N (%)	148 (47.1)		45 (20.7)		41 (35.0)		4 (7.8)		57 (42.9)		66 (22.8)		<0.001^a^
ERI + O, N (%)	45 (14.3)		23 (10.6)		20 (18.0)		4 (7.8)		n.e.		47 (16.2)		n.s.^a^

n.e.: not evaluated; n.s.: not significant.

^a^Pearson's chi square.

^b^GLM adjusted for age and gender.

**Table 3:** Odds ratios (ORs) and 95% confidence intervals (CIs) of job strain and effort/reward imbalance by occupational group (odds for insurance clerks = 1). Adjusted for age and sex.

	Job strain, OR (95% CI)	ERI, OR (95% CI)
Radiologists	6.38 (2.19–18.59)***	11.96 (4.14–34.53)***
Infectious diseases hospital	1.75 (0.59–5.26)	3.00 (1.02–8.77)*
General hospital	5.22 (1.76–15.43)**	6.35 (2.13–18.95)***
Police	2.41 (0.80–7.27)	3.04 (1.03–9.00)*
Mature workers	4.68 (1.52–14.40)**	11.89 (3.91–36.14)***
Insurance clerks	1	1

*P < 0.05; **P < 0.01; ***P < 0.001.
